# Supplementary material for: Bioanalysis of the Ex Vivo Labile PACE4 Inhibitory Peptide Ac-[d-Leu]LLLRVK-Amba in Whole Blood Using Ultra-Performance Liquid Chromatography-Tandem Mass Spectrometry Quantification
Source: Pharmaceutics. 2023 Dec 8;15(12):2745. doi: 10.3390/pharmaceutics15122745 (PMC10747822; doi:10.3390/pharmaceutics15122745)
Supplement: Supplementary file 1 [file pharmaceutics-15-02745-s001.zip › pharmaceutics-2677402-supplementary.pdf]

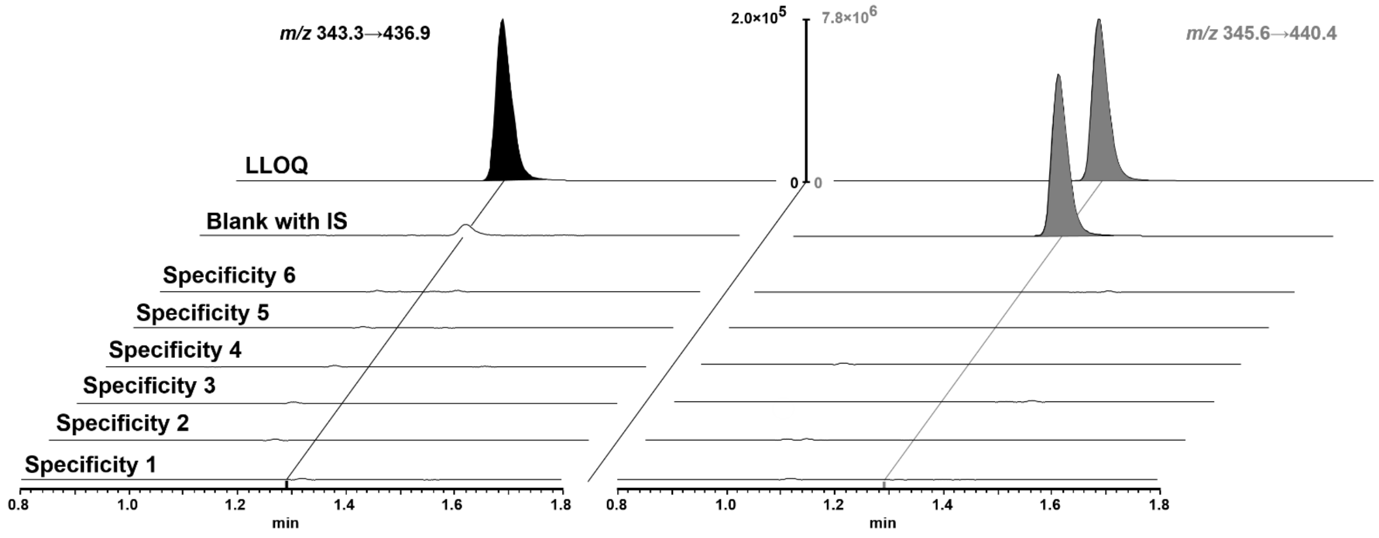

Figure S1: Representative UPLC-MS/MS chromatograms of specificity (blank) samples of Ac-[d-Leu]LLLRVK-amba. The analyte transition is shown in black and the internal standard (IS) transition in grey. The absence of signals at the retention time of the analyte and IS in samples from 6 different mice demonstrates the specificity of the assay. The intensities of the specificity samples were normalized to that of the respective peaks of the lower limit of quantification (LLOQ), which in addition to a blank sample with added IS is shown for comparison.

Table S1: Accuracy and precision data for the autosampler (extract) stability determination

QC level	Accuracy [%]	Precision [% CV]
QC A	98.3	0.9
QC B	99.7	2.4
QC C	97.5	1.2

Table S2: Recovery data of the whole blood validation.

QC	IS normalized recovery [%]
QC A	95
QC B	106
QC C	107

Table S3: Linear regression data of validation batches

Validation batch	R2	Equation
#1	0.997597	0.00354098 × x + 0.00140661
#2	0.996844	0.00346631 × x - 0.00710602
#3	0.993046	0.00352129 × x - 0.00213079

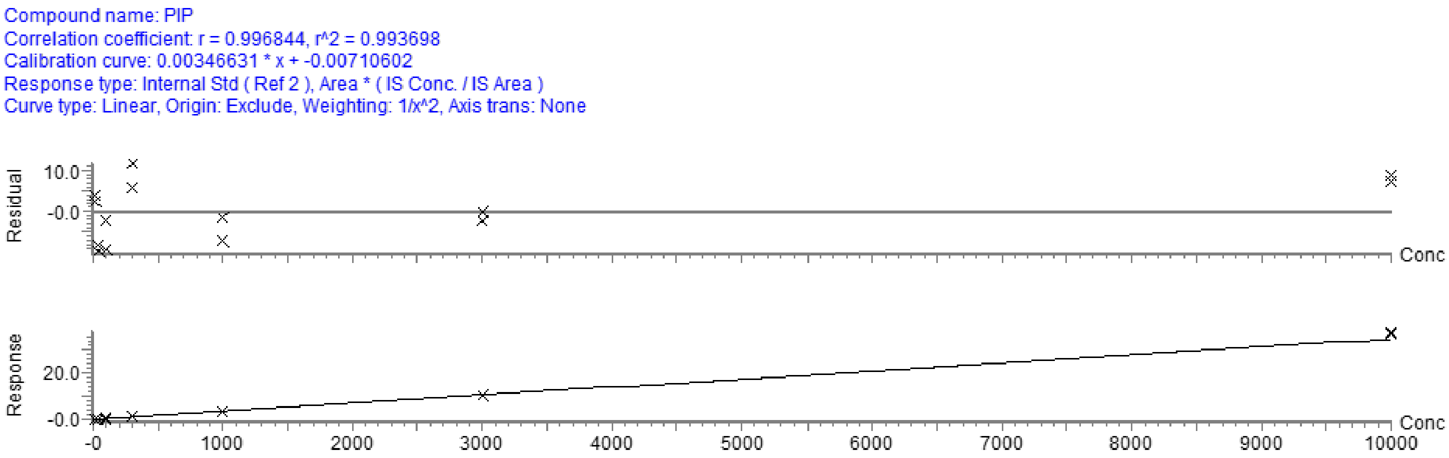

Figure S2: Representative calibration curve of the whole blood validation of Ac-[d-Leu]LLLRVK-amba.

Table S4: Accuracy and precision data for the freeze-and-thaw stability determination

QC level	Accuracy [%]	Precision [% CV]
QC A	91.5	14.0
QC B	91.6	10.2
QC C	98.3	2.5

Further freeze-and-thaw cycles did result in deviations > 15 %. Therefore, blood samples must only be snap-frozen and thawed once before analysis.

Table S5: Internal standard (IS) normalized matrix effect data of the validation.

QC	IS normalized matrix effect [%]
QC A	101
QC B	105
QC C	109
